# Assessment of Ruminal Bacterial and Archaeal Community Structure in Yak (*Bos grunniens*)

**DOI:** 10.3389/fmicb.2017.00179

**Published:** 2017-02-07

**Authors:** Zhenming Zhou, Lei Fang, Qingxiang Meng, Shengli Li, Shatuo Chai, Shujie Liu, Jan Thomas Schonewille

**Affiliations:** ^1^State Key Laboratory of Animal Nutrition, College of Animal Science and Technology, China Agricultural UniversityBeijing, China; ^2^Qinghai Academy of Animal and Veterinary Sciences, Qinghai UniversityXining, China; ^3^Department of Farm Animal Health, Utrecht UniversityUtrecht, Netherlands

**Keywords:** yak, ruminal bacterial community structure, ruminal archaeal community structures, sequencing, feeding regimes

## Abstract

The aim of this study was to determine the microbial community composition in the rumen of yaks under different feeding regimes. Microbial communities were assessed by sequencing bacterial and archaeal 16S ribosomal RNA gene fragments obtained from yaks (*Bos grunniens*) from Qinghai-Tibetan Plateau, China. Samples were obtained from 14 animals allocated to either pasture grazing (Graze), a grazing and supplementary feeding regime (GSF), or an indoor feeding regime (Feed). The predominant bacterial phyla across feeding regimes were *Bacteroidetes* (51.06%) and *Firmicutes* (32.73%). At genus level, 25 genera were shared across all samples. The relative abundance of *Prevotella* in the graze and GSF regime group were significantly higher than that in the feed regime group. Meanwhile, the relative abundance of *Ruminococcus* was lower in the graze group than the feed and GSF regime groups. The most abundant archaeal phylum was *Euryarchaeota*, which accounted for 99.67% of the sequences. Ten genera were detected across feeding regimes, seven genera were shared by all samples, and the most abundant was genus *Methanobrevibacter* (91.60%). The relative abundance of the most detected genera were similar across feeding regime groups. Our results suggest that the ruminal bacterial community structure differs across yak feeding regimes while the archaeal community structures are largely similar.

## Introduction

Yak (*Bos grunniens*) grazing has been the dominant pasture use on the Qinghai-Tibetan plateau, which providing sustenance for pastoralists and products for trade. Given the altitude of this unique habitat (i.e., 4,000 to 5,500 m), yaks are adapted to harsh weather conditions. They traditionally graze in herds, and ingest grasses and/or herbs as their sole source of nutrition. Surveys by the Chinese government and ecologists have identified that most of the Qinghai-Tibetan plateau alpine meadow are overgrazed or degraded (Wang and Fu, 2004; Klein et al., 2007; Harris et al., 2015; Miao et al., 2015). Numerous programs have been initiated to alleviate pasture degradation, including long-term grazing prohibitions and eliminating pastoralism. In addition, other more developed management strategies, such as rotation grazing, supplementary feeding and indoor fence feeding, may also improve the sustainability of grassland use.

There is a general consensus that variation in the ruminal microbial community is responsible for differences in the efficacy and efficiency at which feed is converted to ruminant products. The composition of the ruminal microbial community is sensitive to the diet or feeding regime (Henderson et al., 2015). Therefore, understanding the ruminal microbial community is likely the key to optimizing the production of useful products.

Recent 16S rRNA gene sequencing sequencing has shown the construction of bacterial and methanogenic archaeal communities in the rumen (An et al., 2005; Lozupone and Knight, 2005; Caporaso et al., 2010; Guo et al., 2015; Xue et al., 2016); To date, the yak ruminal microbial community is poorly described. Current data based on clone library sequencing indicates that the yak microbial taxa in the yak rumen are different from those of beef cattle (An et al., 2005; Huang et al., 2012). Given the limited sample size and that only a grazing regime was examined, these data likely represent only a small part of the diverse yak ruminal microbial community. The primary aim of this study was to determine the microbial community of the yak rumen under different feeding regimes by using 16S rRNA gene sequencing. The microbial community of the yak rumen has adapted to specific plant materials due to their unique habitat, and the response of the microbial community to novel feeds is unknown. Thus, we examined animals subject to three distinctive feeding regimes to search for a core microbiome i.e., bacterial and archaeal taxa found across feeding regimes.

## Materials and methods

### Animals and feeding regimes

All animal management and research procedures were approved by the Animal Welfare and Ethical Committee of China Agricultural University (Permit No. DK1318). Experiments were performed in accordance with the Regulations for the Administration of Affairs Concerning Experimental Animals (The State Science and Technology Commission of P. R. China, 1988). Fifteen yak bulls were used to analyze the composition of the ruminal bacterial and archaeal communities. The healthy yak bulls had a body weight of 118 ± 17 kg and were 45 ± 5 months old. They were randomly allocated to three feeding regimes: grazing (graze, *n* = 6), grazing and supplementary feeding (GSF, *n* = 5), or indoor feeding (feed, *n* = 4). The animals allocated to graze regime group grazed in their natural habitat (Mozhugongka county, Lhasa District, Tibet (29°50′13.86″ N, 91°43′46.81 E), about 4,000 m altitude) during summertime. Yaks grazed mixed pastures dominated by *Kobresia pygmaea* and *Stipa purpurea* for majority of the time. Grass samples were collected once every month for determining nutritive quality (from June to October, 2013). Samples were clipped from five 0.25-m^2^ quadrats across the pasture to mimic forage selected by grazing yaks. Then samples were pooled, dried in a forced-air oven at 60°C for 48 h, grounded to pass through a 1-mm sieve, and subsequently analyzed for macronutrient composition. The macronutrient composition of the summer-season pasture was (DM basis): 19.5% crude protein (CP), 3.7% ether extract (EE), 58.6% neutral detergent fiber (NDF), 32.5% acid detergent fiber (ADF), and 8.2% ash. The animals allocated to the feed regime group were individually housed in tie stalls and fed *ad libitum* a mixed forage containing (DM basis): 8.6% CP, 2.9% EE, 68.9% NDF, 35.6% ADF, and 9.45% ash. In addition, the animals were fed 2 kg/day supplemental concentrate with the macronutrient composition (DM basis): 11.1% CP, 5.1% EE, 25.6% NDF, 9.4% ADF, and 11.62% ash. For GSF regime group, yaks were also group-fed daily to provide 2 kg of supplemental concentration when back from pastures at ~1830 h. All yaks had continuous access to high-quality water and trace mineral salt. The whole experiment lasted for 135 days, from June to October, 2013.

### Rumen sampling

All yaks grazing on summer pasture and feed indoor were transported to a commercial slaughter house at the end of the experiment. The animals were stunned and samples (mixture of liquids and solids) were taken from the dorsal, central, and ventral regions of the rumen. Rumen samples were pooled and strained through four layers of cheesecloth to obtain rumen liquids. Aliquots of the pooled rumen liquid samples (~50 mL) were placed on ice, immediately transferred to the laboratory, and stored at −80°C until analysis.

### DNA extraction and sequencing

Metagenomic DNA was extracted from 0.5 g of each homogenized ruminal semi-fluid sample using the repeated bead beating plus column purification (RBB+C) method (Yu and Morrison, 2004) and an oscillator (Precellys 24, Bertin Technology, France). The rotating speed of the oscillator was 5,500 rpm with two circulations (30 s per circulation). The DNA quality was assessed via agarose gel (1%) electrophoresis; metagenomic DNA concentrations were determined with a NanoDrop 2000 Spectrophotometer (Thermo Scientific, USA).

The PCR amplification of the bacterial 16S rRNA V3 region was performed with the primer set 341F/518R (forward primer 341F: 5′-CCT ACG GGA GGC AGC AG-3′; reverse primer 518R: 5′-ATT ACC GCG G CT GCT GG-3′; Zhang et al., 2010; Whiteley et al., 2012; Zhao et al., 2015; Paz et al., 2016). The 5′-end of the reverse primers were fused to an Ion A adaptor plus key sequence and a sample barcode sequence; the forward primers were fused to a truncated Ion P1 adapter sequence. PCR conditions and amplification reaction system have been previously described (Zhao et al., 2015). Amplicons were examined on 2% E-Gel Size SelectTM Agarose Gels and purified with Agencourt AMPure XP Reagent. Library sizes and molar concentrations were determined with a Agilent 2100 Bioanalyzer™ with the Agilent High Sensitivity DNA Kit (Agilent Technologies, Inc., Santa Clara, CA). Emulsion PCR was performed using the Ion OneTouch™ 200 Template Kit v2 DL (Life Technologies, Inc.) according to the manufacturer's instructions. Sequencing of the amplicon libraries was performed on a 318 chip with the Ion Torrent Personal Genome Machine (PGM) system using the Ion PGM™ Sequencing 300 kit (Life Technologies, Inc.).

The archaeal community composition was assessed by sequencing the hypervariable V6~V8 regions of the 16S rRNA gene. DNA was amplified using the universal archaeal primer set Ar915aF/Ar1386R (forward primer Ar915aF: 5′-AGG AAT TGG CGG GGG AGC AC-3′; reverse primer Ar1386R: 5′-GCG GTG TGT GCA AGG AGC-3′; Kittelmann et al., 2013). A unique 10-base error-correcting barcode added to the end of the reverse primer Ar1386R was used for each sample to allow sample multiplexing. The PCR reaction was performed using phusion high-fidelity PCR Mastermix [New England Biolabs (Beijing) LTD., China]. Cycling conditions were: 94°C for 2 min, then 30 cycles of: 94°C for 10 s, 68°C for 20 s, 72°C for 1 min. Amplicons were purified with a Qiaquick PCR purification kit (Qiagen, GmbH) according to manufacturer's instructions. The amplicons from each reaction mixture were pooled at equimolar ratios to generate amplicon libraries. Sequencing was conducted on an Illumina MiSeq 2 × 300 platform according to the protocols described as previous report (Caporaso et al., 2011).

### Sequence processing and analysis

The sequencing data were analyzed using the QIIME pipeline. Sequences were processed for quality control with Fast QC software, and only sequences without ambiguous characters were included in further analysis. FLASH 1.2.7v was used to merge paired-end reads from raw sequencing data (Magoc and Salzberg, 2011). Chimeric sequences were removed using USEARCH sbased on the UCHIME algorithm (Edgar et al., 2011). Samples were excluded from analyses if an insufficient number of sequencing reads were obtained. For bacteria sequencing analysis, one sample was removed from analysis since it had under 14,000 sequences. Thus, 14 samples were used for bacterial community analysis, including four samples from the feed group, six from the graze group, and four from the GSF group. For archaea sequencing analysis, three samples with <8,500 sequencing reads of archaeal 16S rRNA gene were excluded. Thus, 12 rumen liquid samples were used to assess the archaeal community, including four samples from the feed group, three from the graze group, and five from the GSF group. Prior to the calculation of downstream diversity characteristics (i.e., alpha and beta diversity), all samples were subsampled to equal size. The microbial diversity was analyzed using QIIME 1.7.0v (Caporaso et al., 2010) with Python scripts. The sequences were clustered into Operational Taxonomic Unit (OTUs) using the *de novo* OTU picking protocol with a 97% similarity threshold. Representative sequences of OTUs were aligned to the Greengenes database (version 13_8) for bacterial and archaeal 16S rRNA genes. Alpha diversity analysis (i.e., observed species, Chao1, Shannon-Weiner, and Simpson's indices) were generated and jackknifed beta diversity, including those based on both unweighted and weighted Unifrac distances, were visualized using principal coordinate analysis (PCoA; Lozupone and Knight, 2005). Sequence number, sample coverage, unique OTUs, sample richness, sample diversity, phylum relative abundance, and genus were evaluated using the generalized linear model (GLM) procedure in SAS (version 9.1.3; SAS Institute Inc., Cary, NC, USA). Means were separated by using the Student-Newman-Keuls test (SNK). After the multiple testing analyses, false discovery rate (FDR) adjusted *p*-values, also called *q*-values, were computed by using the QVALUE software (version 2.6.0) in R package (version 3.1.0). The *q*-value of FDR <0.05 represents significant difference in microbe relative abundances between the three feeding regimes.

## Results

### Sequencing and general bacterial community composition

We generated a total of 468,400 sequences from 14 samples that passed all filtering metrics; the average length across all samples was about 190 bp. The number of sequences per animal ranged from 14,082 (GSF group) to 46,450 (graze group), with mean of 33,457 sequences. A total of 43,525 OTUs were detected from 14 samples at 3% dissimilarity. Each sample had an average of 9,473 OTUs. Richness estimates and diversity indices were developed after normalization to 14,000 sequences (Table S1).

**Figure 1:**
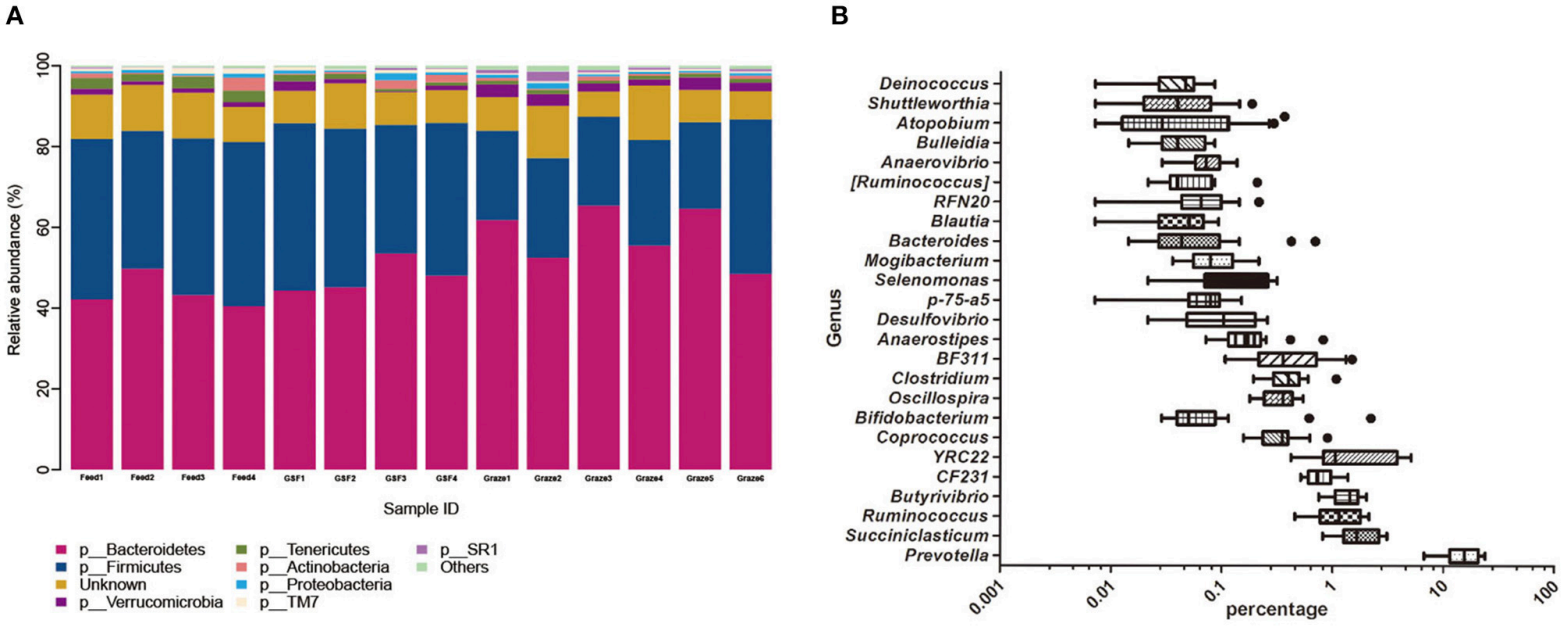
**Dominant bacterial phylum in individual samples and the shared bacterial genera across ruminal samples. (A)** The bacterial taxonomic composition of individual yak ruminal samples at the phylum level. **(B)** The relative abundance of shared bacterial genera across yak ruminal samples in shown via a box plot of the relative abundance of bacterial genera shared by all samples. Percentage is shown on the X-axis. The boxes represent the interquartile range (IQR) between the first and third quartiles (i.e., 25th and 75th%, respectively), and the vertical line inside the box defines the median. Whiskers represent the lowest and highest values within 1.5 times the IQR from the first and third quartiles. Samples with a relative abundance for a given genus exceeding those values are represented as points beside the boxes. The groups are: indoor feeding regime (feed), pasture grazing regime (graze), and grazing, and supplementary feeding regime (GSF).

We found 19 distinct phyla across feeding regimes; the most abundant were *Bacteroidetes* (51.06%) and *Firmicutes* (32.73%). The top eight phyla, which had high relative abundances and were prevalent in all samples (Figure 1A), accounted for nearly 90% of all sequences. The less abundant phyla included *Verrucomicrobia* (1.73%), *Tenericutes* (1.43%), *Actinobacteria* (0.94%), *Proteobacteria* (0.76%), TM7 (0.67%), and SR1 (0.47%). The other known phyla with relative abundances <0.4% accounted for 0.66% of all sequence data, while the remaining sequences that were unclassified at the phylum level accounted for 9.54%. We detected 120 distinct genera across all feeding regimes, and 25 genera were present in all samples (Figure 1B); this demonstrates the variability in abundance across samples. The top nine genera had relative abundances above 0.04%. The predominant genera across all samples were *Prevotella* (15.81%), YRC22 (2.08%), *Succiniclasticum* (1.87%), *Butyrivibrio* (1.40%), and *Ruminococcus* (1.25%). Minor genera, such as CF231, BF311, *Clostridium*, and *Bifidobacterium* accounted for 0.81, 0.53, 0.43, and 0.25%, of relative abundance, respectively. The other known genera accounted for 2.74% of sequences, while sequences that were unclassified at the genus level accounted for 72.85% of sequences.

### Sequencing and general archaeal community composition

**Figure 2:**
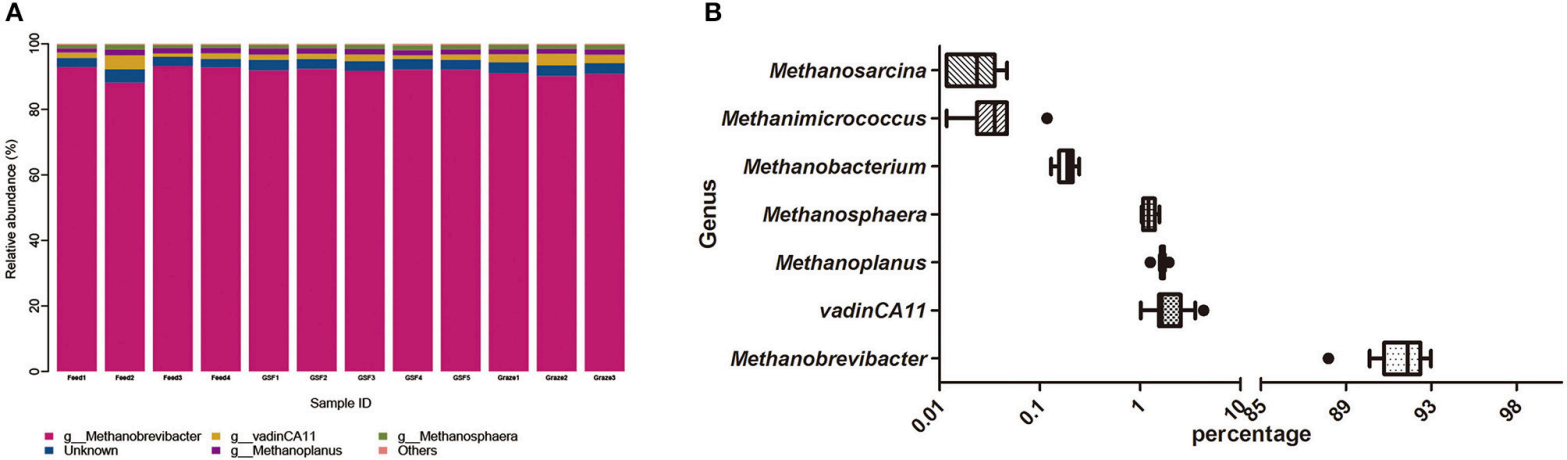
**Dominant archaeal genera in individual sample and the shared archaeal genera across ruminal samples. (A)** Archaeal taxonomic composition of individual yak ruminal samples at the genus level. **(B)** Relative abundance of shared archaeal genera across yak ruminal samples, showen as a box plot of relative bacterial genera abundance shared by all samples with percentage on the *X*-axis. The boxes represent the interquartile range (IQR) between the first and third quartiles (i.e., 25th and 75th%, respectively), and the vertical line inside the box defines the median. The whiskers represent the lowest and highest values within 1.5 times the IQR of the first and third quartiles, respectively. Samples with a relative abundance exceeding those values for a given genus are represented as points besides the boxes. The groups are: indoor feeding regime (feed), pasture grazing regime (graze), and grazing and supplementary feeding regime (GSF).

We generated 226,711 archaeal sequences from 12 samples with an average length of 470 bp (Table S2). This was an average of 18,892 sequences, or 851 OTUs per animal. Richness estimates and diversity indices were developed after normalization to 8,500 sequences. Four distinct phyla were found across feeding regimes. *Euryarchaeota* was the most abundant phylum in all samples, accounting for 99.67% of the all archaeal sequences (Figure 2A). *Crenarchaeota* accounted for 0.11% of archaeal sequences, while the remaining sequences (0.12% of sequences) could not be classified at the phylum level. We detected 10 distinct genera across feeding regimes, and there were seven genera that were shared by all samples (Figure 2B). The most abundant genus was *Methanobrevibacter* (91.60%). vadinCA11, *Methanoplanus, Methanosphaera, Methanobacterium, Methanimicrococcus*, and *Methanosarcina* accounted for 2.07, 1.65, 1.24, 0.19, 0.04, and 0.02% of archaeal sequences, respectively. The remaining 3.17% of sequences were unclassified at the genus level.

### Effects of feeding regime on the bacterial community

**Figure 3:**
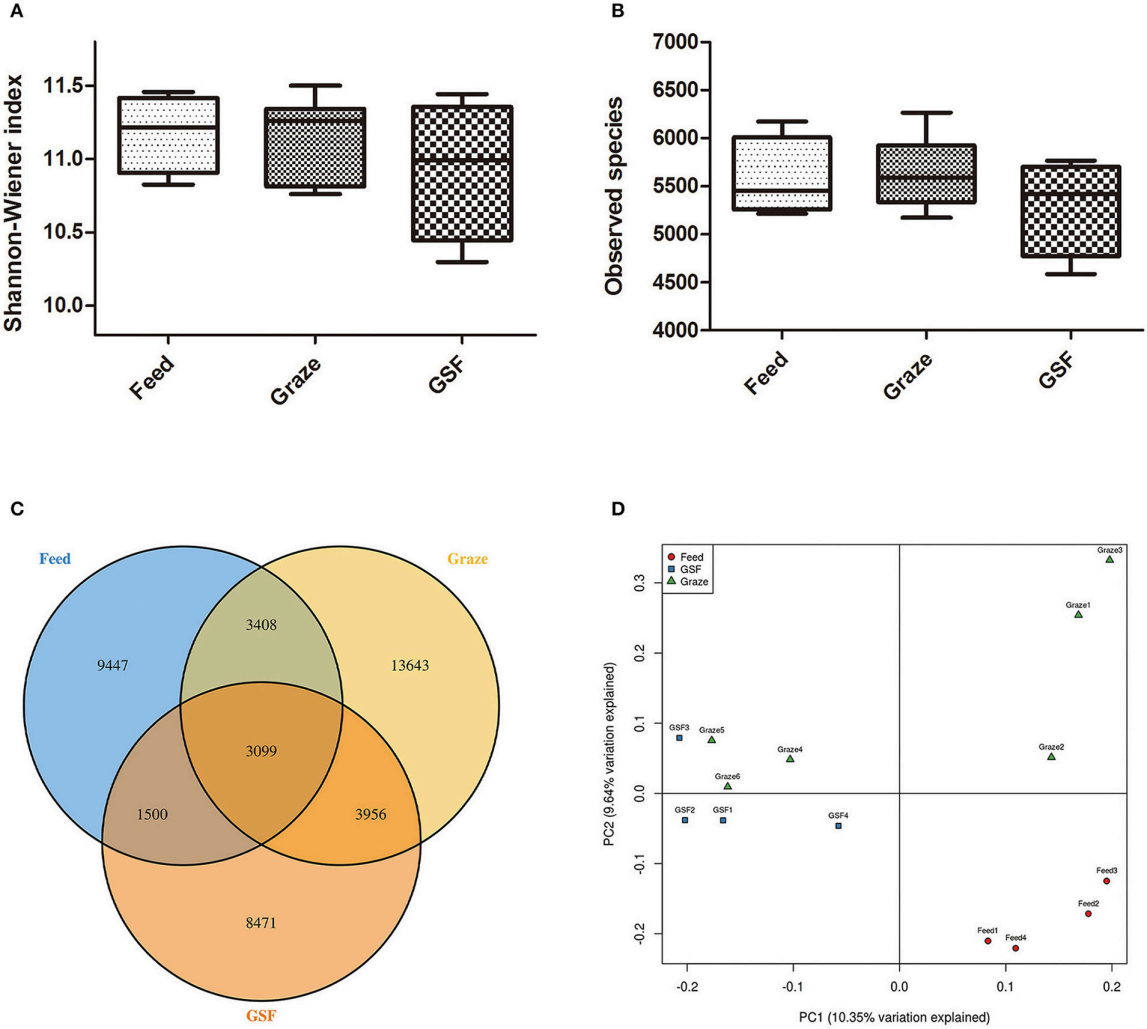
**Differences in bacterial community diversity, richness, and OTUs between feeding regimes. (A)** Shannon-Weiner diversity in yak ruminal samples. **(B)** Observed species in yak ruminal samples. **(C)** A venn diagram showing the different and similar OTUs between feeding regimes. **(D)** A principal coordinate analysis (PCoA) of the yak ruminal microbiota from the three feeding groups. The PCoA plots were constructed using the unweighted UniFrac method. The groups are: indoor feeding regime (feed), pasture grazing regime (graze), and grazing and supplementary feeding regime (GSF).

The Shannon-Weiner and observed species indices were similar (*q* > 0.05) between the three feeding regimens (Figures 3A,B). The similarity in archaeal OTUs between feeding regimes was visualized in a Venn diagram (Figure 3C). A thermal double dendrogram of the 80 most abundant bacterial OTUs illustrated that GSF regime samples could not grouped from other two treatments (Figure S1); while indoor feed regime samples could clearly separate from that of pasture graze regime. The PCoA analysis showed the relationships between the microbiome community structures (Figure 3D). There was no obvious dividing line and the two principal components covered 21.72% of the variation; furthermore, samples from the graze regime group could not be distinguished from samples from the GSF regime group. Interestingly, the microbiome of the indoor feed group was distinct from that of the graze group.

The relative abundance of bacterial taxa were used to describe the impact of feeding regimes on bacterial populations. At phylum level (Table 1), the ruminal microbiome from the graze group had significantly higher relative abundance of *Bacteroidetes* than the samples from the feed and GSF regime groups (*q* = 0.02). While there is a lower relative abundance in *Firmicutes* in graze regime group (*q* = 0.02). The graze regime group had higher relative abundance of *Verrucomicrobia* compared with the feed and GSF regime groups (*q* = 0.04). The feed regime group had higher relative abundances of *Tenericutes* and TM7 than the graze regime group (*q* < 0.01 and *q* = 0.04, respectively). We found no differences in the rest of the phyla (*q* > 0.05). At genus level (Table 2), the relative abundances of *Prevotella* in the graze and GSF regime groups were significantly higher than in the feed regime group (*q* = 0.03). The relative abundance of *Ruminococcus* in the graze regime group was lower than in the feed and GSF regime group (*q* = 0.02). No significant differences in community structure were observed in the rest of the genera (*q* > 0.05). These data suggest that the bacterial community structure differs between the three feeding regimes.

**Table 1:** **Effect of feeding regime on phylum-level diversity (% of total sequences) in the rumen bacterial community**.

**Phylum**	**Feeding regime^1^**			**SEM**	***q*-value^2^**
	**Feed**	**GSF**	**Graze**		
*Bacteroidetes*	43.91^b^	47.76^b^	58.03^a^	2.27	0.02
*Firmicutes*	38.32^a^	37.60^a^	25.76^b^	2.06	0.02
Unknown	10.58	8.84	9.31	0.96	0.63
*Verrucomicrobia*	1.16^b^	1.19^b^	2.46^a^	0.28	0.04
*Tenericutes*	2.56^a^	1.10^b^	0.90^b^	0.15	<0.01
*Actinobacteria*	1.22	1.17	0.61	0.37	0.64
*Proteobacteria*	0.67	0.89	0.72	0.17	0.73
TM7	0.99^a^	0.69^a^^,^ ^b^	0.45^b^	0.10	0.04
SR1	0.14	0.22	0.86	0.22	0.16
Others	0.45	0.53	0.88	0.11	0.11

1 *Feed, indoor feeding regime; Graze, pasture grazing regime; GSF, grazing and supplementary feeding regime*.

2 *q-value: false discovery rate (FDR) adjusted p-value*.

a, b *Values in the same row with different superscripts are significantly different (q < 0.05)*.

**Table 2:** **Effect of feeding regime on genus-level diversity (% of total sequences) in the rumen bacterial community**.

**Phylum**	**Genus**	**Feeding regime^1^**			**SEM**	***q*-value^2^**
		**Feed**	**GSF**	**Graze**		
*Bacteroidetes*	*Prevotella*	9.64^b^	15.64^a^	20.03^a^	1.46	0.03
*Bacteroidetes*	YRC22	0.66	2.06	3.04	0.59	0.26
*Firmicutes*	*Succiniclasticum*	2.09	2.24	1.49	0.29	0.36
*Firmicutes*	*Butyrivibrio*	1.57	1.52	1.20	0.17	0.43
*Firmicutes*	*Ruminococcus*	1.79^a^	1.40^a^	0.78^b^	0.16	0.02
*Bacteroidetes*	CF231	0.83	0.71	0.86	0.11	0.68
*Bacteroidetes*	BF311	0.23	0.53	0.73	0.16	0.39
*Firmicutes*	*Clostridium*	0.39	0.55	0.38	0.09	0.53
*Actinobacteria*	*Bifidobacterium*	0.73	0.07	0.04	0.22	0.36
Others	Others	2.86	3.24	2.32	0.31	0.34

1 *Feed, indoor feeding regime; Graze, pasture grazing regime; GSF, grazing and supplementary feeding regime*.

2 *q-value: false discovery rate (FDR) adjusted p-value*.

a, b *Values in the same row with different superscripts are significantly different (q < 0.05)*.

### Effects of feeding regime on the archaeal community

**Figure 4:**
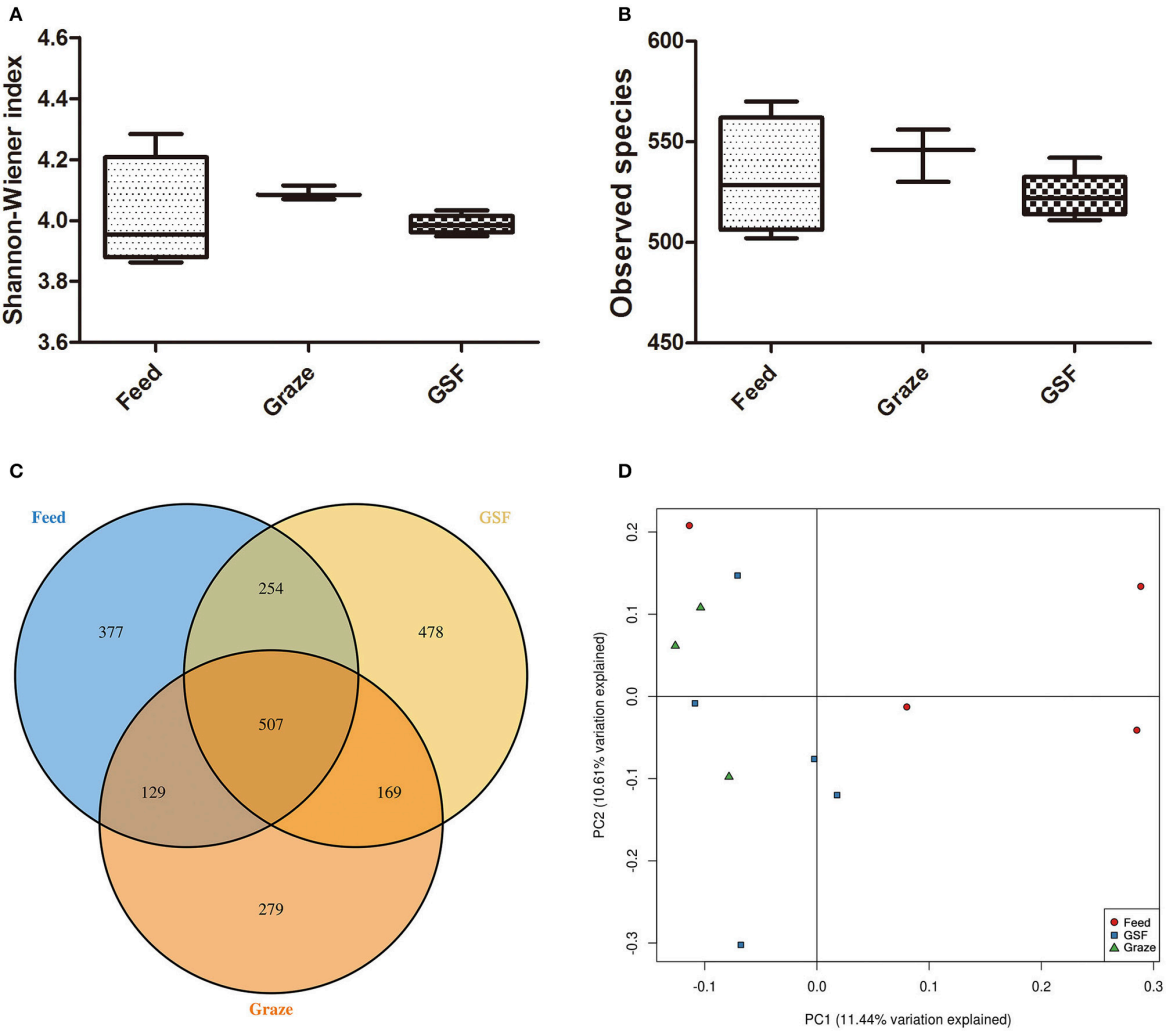
**Differences in archaeal community diversity, richness, and OTUs between the feeding regimes. (A)** Shannon-Weiner diversity in yak ruminal samples. **(B)** Observed species in yak ruminal samples. **(C)** A Venn diagram showing the number of differences and shared OTUs between the feeding regimes. **(D)** Principal Coordinate Analysis (PCoA) of archaeal community structures of the yak ruminal microbiota of the three feeding groups. The PCoA plots were constructed using the unweighted UniFrac method. The groups are: indoor feeding regime (feed), pasture grazing regime (graze), and grazing and supplementary feeding regime (GSF).

Analyses of archaeal 16S rRNA gene fragments revealed no differences in diversity between the feeding regimes (Figures 4A,B) by the Shannon-Weiner index or the observed species analyses. The similarity in archaeal OTUs between feeding regimes was visualized in a Venn diagram (Figure 4C). The number of archaeal OTUs in the feed regime group was similar to that of the graze and GSF regime groups (Table S2). A thermal double dendrogram of the 80 most abundant archaeal OTUs illustrated that samples in the same regime were not clearly grouped (Figure S2); this implies that the archaeal communities are similar between feeding regimes. Furthermore, the overall archaeal community structure analyzed by PCA analysis based on unweighted UniFrac method revealed no obvious differences between regime groups (Figure 4D). The relative abundance of the most detected genera were similar across feeding regime groups (*q* > 0.05; Table 3).

**Table 3:** **Effect of feeding regime on genus-level diversity (% of total sequences) in the rumen archaeal community**.

**Phylum**	**Genus**	**Feeding regime^1^**			**SEM**	***q*-value^2^**
		**Feed**	**GSF**	**Graze**		
*Euryarchaeota*	*Methanobrevibacter*	91.74	92.05	90.65	0.81	0.42
*Euryarchaeota*	vadinCA11	2.14	1.53	2.85	0.53	0.67
*Euryarchaeota*	*Methanoplanus*	1.56	1.73	1.63	0.09	0.70
*Euryarchaeota*	*Methanosphaera*	1.18	1.25	1.30	0.11	0.84
*Euryarchaeota*	*Methanobacterium*	0.19	0.18	0.19	0.02	0.95
*Euryarchaeota*	*Methanimicrococcus*	0.03	0.05	0.04	0.02	0.59
*Euryarchaeota*	*Methanosarcina*	0.01	0.03	0.02	0.01	0.28

1 *Feed, indoor feeding regime; Graze, pasture grazing regime; GSF, grazing and supplementary feeding regime*.

2 *q-value: false discovery rate (FDR) adjusted p-value*.

*^a, b^ Values in the same row with different superscripts are significantly different (q < 0.05)*.

## Discussion

Qinghai-Tibetan plateau pasture generally have high contents of complex plant polysaccharides that cannot be digested by yaks alone. Ruminal microbes provide the enzymes to partially convert these polysaccharides and other plant components to volatile fatty acids and microbial biomass, which are major metabolic substrates that can then be utilized by the host. This symbiotic relationship between ruminal microbes and yaks enables the animal to convert indigestible plant materials to high valued products, such as meat and milk.

Sequencing allows for identification of an enormous number of bacterial and archaeal taxa, which giving a broad description of the ruminal community. In our study, 25 genera were shared across feeding regimes. While there was some variation in relative abundance between taxa in ruminants, such as beef cattle, the core rumen microbiome consisted of 10 distinct bacterial taxa (Petri et al., 2013); meanwhile, lactating dairy cows had a core rumen microbiome consisting of 32 genera (Jami and Mizrahi, 2012). Recent 16S rRNA gene sequencing has shown the community construction of yak ruminal bacteria and methanogenic archaea (Guo et al., 2015; Xue et al., 2016). However, given the limited number of animals sampled, and that only one type of feeding regime (grazing) was examined, these data likely only represent only a part of the diverse yak ruminal community. It is impossible to infer the core microbiome in the yak rumen. In fact, we also detected total 122 genus across 14 samples, the amount was similar to the amount seen in the pooled three rumen samples (Chen et al., 2015). Compared with the community data of 194 clones library (An et al., 2005), the bacterial community had similar predominant members, including the *Prevotella, Succiniclasticum, Butyrivibrio, Ruminococcus*, and *Clostridium* genera. We show similar bacterial and archaeal communities as the recently-described rumen and camelid foregut microbial community from 32 animal species (Henderson et al., 2015). Given the inherent differences in methodology between studies, these bacterial genera may represent the highly conserved “core microbiome” of the yak rumen.

Similar to previous ruminant studies (Petri et al., 2013; Jami et al., 2014; Kim et al., 2014; Pitta et al., 2014b; Jewell et al., 2015), *Bacteroidetes* and *Firmicutes* were the predominant phyla regardless of feeding regime. The yak ruminal community was similar to that of steers grazing on winter wheat forage or pasture, which were also dominated by *Bacteroidetes* (59 ~ 77%) and *Firmicutes* (20 ~ 33%; McCann et al., 2014; Pitta et al., 2014b). This is also similar to buffalo fed high forage diets (*Bacteroidetes* ranged from 48.4 to 55.9% and *Firmicutes* from 18.0 to 27.8%; Pitta et al., 2014a). Within the *Bacteroidetes* phylum, high relative abundance of *Prevotella* has been found with different types of diets, such as diets based on forage, browse, or concentrate (Petri et al., 2013; Pinloche et al., 2013; Kim et al., 2014; Rey et al., 2014; Zhang et al., 2014; Jewell et al., 2015). Our results also showed that *Prevotella* seem to be highly abundant in all these types of feeding regimes, which may suggest substantial metabolic diversity in the rumen ecosystem.

Sequencing revealed that the feeding regime had effect on the composition and biodiversity of the ruminal bacterial community. The relative abundance of *Firmicutes* in the feed group was noticeable higher than in the graze groups, which indicates a core microbiome associated with digestion of a more recalcitrant fiber in the indoor feeding regime. The graze and GSF regime groups had higher relative abundances of *Prevotella* than the feed group; this indicates that *Prevotella* species have diverse functions in the rumen. However, feeding regime had a similar effect on the *Ruminococcus* genus of the *Firmicutes*. Although representing a very small fraction, its present in all samples could indicate that *Ruminococcus* occupies a unique and important ecological niche in the rumen. In fact, some species within this genus, such as *Ruminococcus flavefaciens, Ruminococcus albus*, and *Ruminococcus bromii*, have the capacity to degrade fiber in the feed. Given that many bacteria within the rumen only utilize specific substrates, the type of feeding regime is likely to have a substantial impact on the composition of the ruminal microbiome. The diversity of and discrimination between feeding regimes is likely driven by specific forage characteristics and diet composition. Indoor feeding diets have greater amounts of recalcitrant carbohydrate, mainly from corn stalk. Meanwhile, the summer-season pasture is characterized by relatively high crude protein content, high ether extract, and low neutral detergent fiber levels. In the summer season, animals feeding via the grazing system will consume more digestible grass compared with those feeding via the indoor feeding regime. Cluster analysis of the sequencing profiles confirmed the differentiation between communities associated with pasture grazing and indoor feeding. These differences were apparent even though 72.85% of sequences could not be classified at the genus level. For this reason, we must exhibit caution when interpreting the effects of feeding regime on the ruminal microbial community. In addition, other members of the ruminal microbiome community, such as fungi and protozoa should be investigated to further elucidate these results.

Feeding regimes had no effect on the relative abundance of dominant archaeal taxa. *Methanobrevibacter* was dominant in rumen samples from all the feeding regimes, and represented ≥90% of all archaeal sequences. Previous reports have shown that *Methanobrevibacter* is dominant archaeal genus in the rumen of impala (*Aepyceros melampus*; Cersosimo et al., 2015) deer (*Capreolus pygargus*; Li et al., 2013, 2014), water buffaloes (*Bubalus bubalis*; Franzolin et al., 2012), and dairy cows (Hook et al., 2011; Danielsson et al., 2012). Within this genus, *Methanobrevibacter ruminantium, Methanobrevibacter thaueri, Methanobrevibacter smithii*, and *Methanosphaera stadtmanae* are the most common methanogens in the bovine rumen (Jarvis et al., 2000; Whitford et al., 2001; Wright et al., 2007; Zhou et al., 2009). Although the *Methanocorpusculum* genus was only detected in the GSF sample, it is difficult to propose a link between this specific genus and the GSF feeding regime since it was only found in one sample and was at a low relative abundance (0.01%) in that sample. *Methanocorpusculum* has previously been reported to attach to the hydrogenosomes of ciliate and capture H_2_ escaping from hydrogenosomes (Finlay et al., 1993). *Methanoculleus* and *Methanolobus* also observed, but the results were not consistent across all samples from a given group. Other archaeal genera observed in the rumen samples were vadinCA11, *Methanoplanus, Methanosphaera, Methanobacterium, Methanimicrococcus*, and *Methanosarcina*. Low relative abundance microbes may play important roles in different environments. Therefore, even though these genera accounted for only 9% of all sequences, they may play an important role in yak feed efficiency and methane emissions.

In summary, we investigated the bacterial and archaeal community structures of the yak rumen using high-through sequencing. We found that the type of feeding regime influences the bacterial diversity and microbiome composition, as several ruminal taxa varied significantly across feeding regimes. However, there was less effect on the archaeal community structure. *Bacteroidetes* and *Firmicutes* were the most abundant bacterial phyla in the rumen, and 25 genera were shared across all ruminal samples. The most abundant archaeal genus was *Methanobrevibacter*, and seven archaeal genera were shared across all samples. Additional work is needed to continue to elucidate host-microbe interactions in yaks.

## Author contributions

ZZ designed the experiment and wrote the manuscript; LF and QM conducted the research; SLiu, SC, JS, and SLi reviewed the manuscript. ZZ had primary responsibility for the final content. All authors read and approved the final manuscript.

### Conflict of interest statement

The authors declare that the research was conducted in the absence of any commercial or financial relationships that could be construed as a potential conflict of interest.
