# Supplementary material for: Assessment of Ruminal Bacterial and Archaeal Community Structure in Yak (Bos grunniens)
Source: Front Microbiol. 2017 Feb 7;8:179. doi: 10.3389/fmicb.2017.00179 (PMC5293774; doi:10.3389/fmicb.2017.00179)
Supplement: Supplementary file 1 [file Table1.PDF]

S1 Table. Individual Yak bulls for bacterial unique OTUs, richness estimates, and diversity indices within the rumen content.

SampleID	SeqsNum	OTUsNum	EvenSeqsNum	EvenOTUsNum	ACE	simpson	shannon	PD_whole_tree
Feed1	35581	9630	14000	5511	14588.05	0.998799	11.28755	537.2487
Feed2	40307	10341	14000	5395	15527.61	0.995129	10.82566	532.9342
Feed3	36566	9470	14000	5213	13996.96	0.998596	11.1419	512.3313
Feed4	45477	13180	14000	6173	18640.48	0.99834	11.45828	590.0272
Graze1	42288	10849	14000	5387	15731.59	0.99586	10.82938	517.3268
Graze2	28259	8922	14000	5714	15467.97	0.997938	11.29038	540.4055
Graze3	46450	12138	14000	5808	16746.02	0.997598	11.28566	543.5483
Graze4	35251	11307	14000	6266	18390.93	0.998616	11.49918	577.7009
Graze5	23949	7368	14000	5173	13911.66	0.996084	10.76066	488.7122
Graze6	35191	9774	14000	5466	15782.73	0.99882	11.23635	524.4167
GSF1	14082	5345	14000	5326	12917.17	0.995916	10.89049	502.8674
GSF2	34178	7982	14000	4585	12834.81	0.995844	10.29673	455.9407
GSF3	29971	8869	14000	5518	15527.28	0.997971	11.09416	526.6116
GSF4	20850	7451	14000	5767	15247.06	0.998996	11.44219	544.1519
