# Supplementary material for: Assessment of Ruminal Bacterial and Archaeal Community Structure in Yak (Bos grunniens)
Source: Front Microbiol. 2017 Feb 7;8:179. doi: 10.3389/fmicb.2017.00179 (PMC5293774; doi:10.3389/fmicb.2017.00179)
Supplement: Supplementary file 2 [file Table2.PDF]

S2 Table. Individual Yak bulls for archaeal unique OTUs, richness estimates, and diversity indices within the rumen content.

Sample ID	SeqsNum	OTUsNum	EvenSeqsNum	EvenOTUsNum	ACE	simpson	shannon	PD_whole_tree	chao1	observed_species
Feed1	32309	1211	8500	538	1544.56	0.833548	3.978861	13.1288	1276.907	538
Feed2	8634	574	8500	570	1365.789	0.850413	4.28401	14.21955	1338.718	570
Feed3	23718	969	8500	519	1576.572	0.818999	3.862798	12.31854	1451.552	519
Feed4	26227	1072	8500	502	1443.279	0.834056	3.931025	12.03112	1397.172	502
Graze1	10534	632	8500	556	1485.487	0.840263	4.115541	13.97717	1333.247	556
Graze2	13665	735	8500	530	1455.311	0.839666	4.084865	13.46574	1200.241	530
Graze3	12273	697	8500	546	1544.238	0.834504	4.070356	13.42043	1248.391	546
GSF1	11770	661	8500	542	1661.642	0.833623	3.984159	13.3575	1599.851	542
GSF2	15046	761	8500	523	1402.287	0.826399	3.947954	13.16264	1301.538	523
GSF3	23615	1002	8500	522	1447.656	0.839152	4.032513	13.11709	1282.769	522
GSF4	9022	537	8500	517	1490.423	0.836262	3.996682	13.46029	1312.8	517
GSF5	39898	1362	8500	511	1451	0.832634	3.975245	13.36675	1404.879	511
