# Supplementary material for: Assessment of Ruminal Bacterial and Archaeal Community Structure in Yak (Bos grunniens)
Source: Front Microbiol. 2017 Feb 7;8:179. doi: 10.3389/fmicb.2017.00179 (PMC5293774; doi:10.3389/fmicb.2017.00179)
Supplement: Supplementary file 3 [file Image1.PDF]

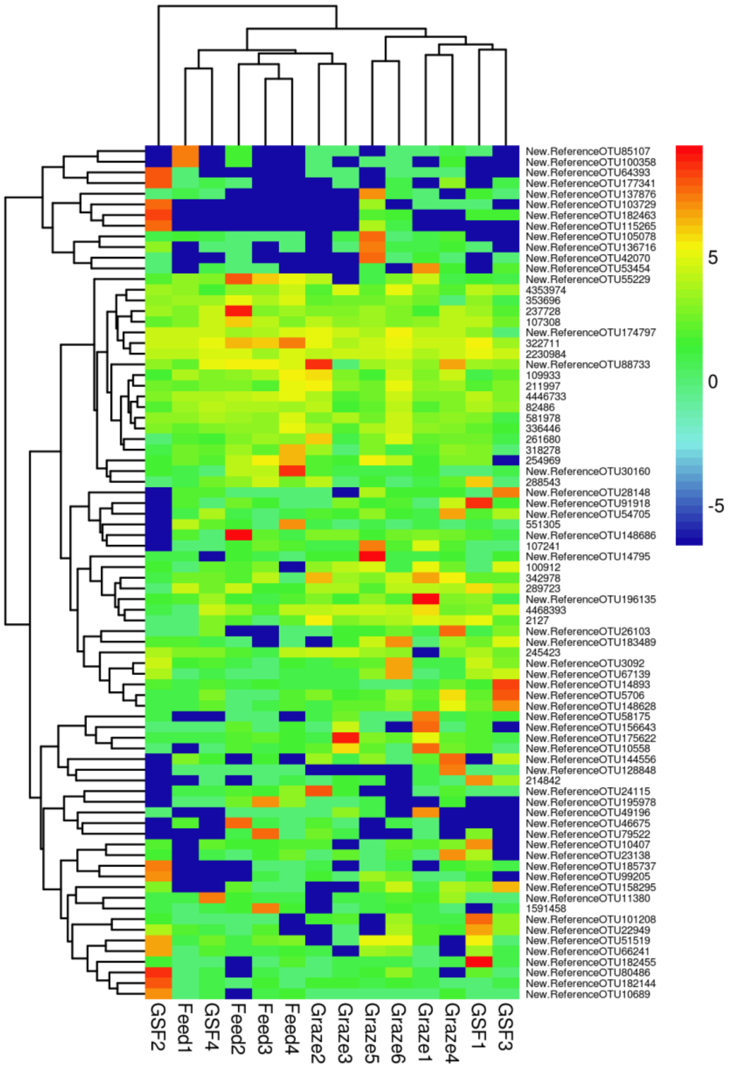

S1 Fig. Thermal double dendrogram of the most abundant bacterial operational taxonomic units (top 80 OTUs).
