# Supplementary figures and images for: Assessment of Ruminal Bacterial and Archaeal Community Structure in Yak (Bos grunniens)
Source: Front Microbiol. 2017 Feb 7;8:179. doi: 10.3389/fmicb.2017.00179 (PMC5293774; doi:10.3389/fmicb.2017.00179)

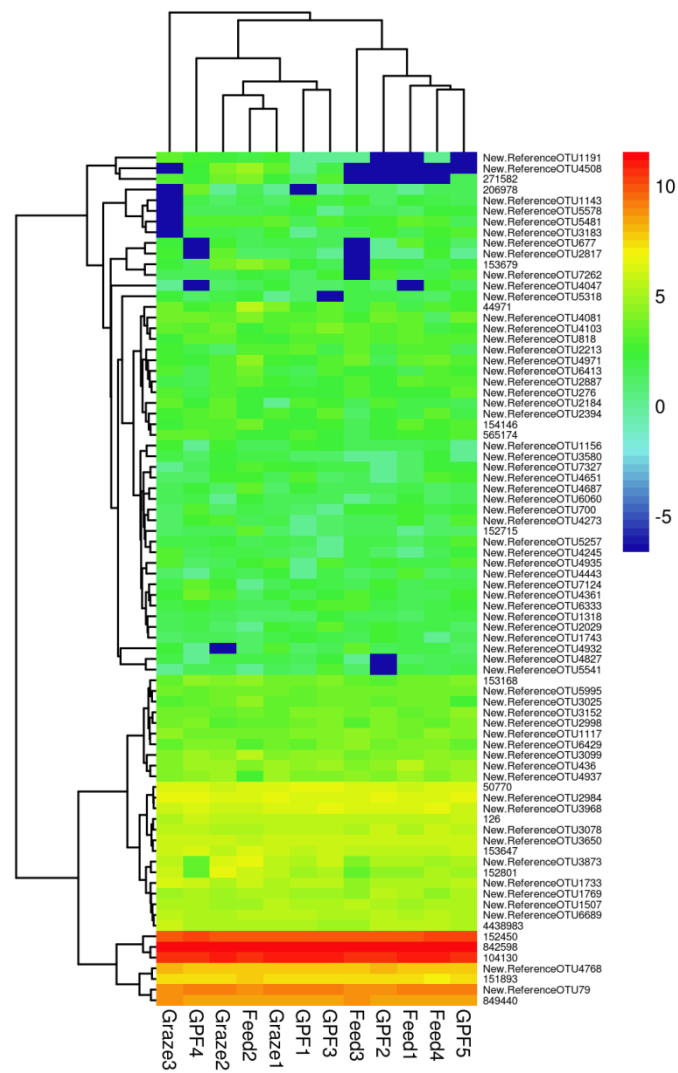

OTUs).

Supplement: Supplementary file 4 [file Image2.PDF]
